# Electro-Therapeutics

**Published:** 1889-06

**Authors:** 


ELECTRO-THERAPEUTICS, OR ELECTRICITY IN ITS RELATIONS TO MEDICINE AND SURGERY. By William Harvey King, M. D. Pages, 152. New York: A. L. Chatterton & Co. 1889.

The subject of Electro-Therapeutics has attracted the attention of the profession, during the past few years, to a very great extent. That electricity *has* a place in therapeutics none will deny; but to accurately locate this place is a difficult question at present. As Dr. King says (p. 74), "Electricity is not a cure-all, but has its special sphere of action and indications, the same as any other remedy; and the more closely these indications are studied and the treatment applied accordingly, the surer will be the success." This is true of all therapeutic agents, and if Dr. King can teach us the special sphere or the true indications for the proper use of electricity he will certainly confer a favor upon the profession. Dr. King, as do all other electropaths, well describes his apparatus, and shows the motor points for the proper application of the electrodes, but (as it seems to us) fails to give indications for the use of the electricity as a proper homœopathic remedy.

